# Exploring Factors Influencing Scenarios Evolution of Waste NIMBY Crisis: Analysis of Typical Cases in China

**DOI:** 10.3390/ijerph18042006

**Published:** 2021-02-19

**Authors:** Ling He, Qing Yang, Xingxing Liu, Lingmei Fu, Jinmei Wang

**Affiliations:** 1 School of Management, Wuhan University of Technology, Wuhan 430070, China; heling@whut.edu.cn (L.H.); yangq@whut.edu.cn (Q.Y.); mlsdb@whut.edu.cn (L.F.); wangjinmei@whut.edu.cn (J.W.); 2 School of Safety Science and Emergency Management, Wuhan University of Technology, Wuhan 430070, China

**Keywords:** waste NIMBY crisis, ground theory, dynamic Bayesian network, scenario evolution, probability analysis, node variables

## Abstract

As the impact factors of the waste Not-In-My-Back Yard (NIMBY) crisis are complex, and the scenario evolution path of it is diverse. Once the crisis is not handled properly, it will bring adverse effects on the construction of waste NIMBY facilities, economic development and social stability. Consequently, based on ground theory, this paper takes the waste NIMBY crisis in China from 2006 to 2019 as typical cases, through coding analysis, scenario evolution factors of waste NIMBY crisis are established. Furtherly, three key scenarios were obtained, namely, external situation (E), situation state (S), emergency management (M), what is more, scenario evolution law of waste NIMBY crisis is revealed. Then, the dynamic Bayesian network theory is used to construct the dynamic scenario evolution network of waste NIMBY crisis. Finally, based on the above models, Xiantao waste NIMBY crisis is taken as a case study, and the dynamic process of scenario evolution network is visually displayed by using Netica. The simulation results show that the scenario evolution network of Xiantao waste NIMBY crisis is basically consistent with the actual incident development process, which confirms the effectiveness and feasibility of the model.

## 1. Introduction

Municipal solid waste (MSW) incineration has been strongly promoted in China for more than ten years, in order to eliminate the contradiction between the growth of MSW and the shortage of construction land in densely populated cities [1]. The rapid expansion of MSW incineration causes the Not-In-My-Back Yard (NIMBY) syndrome [2]. In NIMBY incidents, each stakeholder possesses their own interests and holds different attitudes towards NIMBY facilities. Only by fully grasping the interests, the impacts and the relationships that exists among various stakeholders can governments coordinate to achieve a win-win outcome. Otherwise, there will be socially inequitable because a minority group bearing a disproportionate share of the negative externalities of these NIMBY facilities [3].

Social impacts of NIMBY facilities are complex in nature. Once believed that the living environment, personal health, and safety are compromised, these facilities are often confronted with the opposition and resistance from the public, leading to the severely social conflicts [4]. Rapid urbanization in China brings a large number of urban public facilities and infrastructures, which benefit the development of cities as a whole but generally have adverse external impacts on the proximate residents [5]. Under such circumstance, these projects may cause public concerns and lead to the public protest, even social conflicts [6].

When there is a conflict between the public interest and the private interest, if the citizens feel that their own interests are being harmed or are unfairly treated, they will take irrational ways to fight, such as mass demonstrations. The effect of the “not in my backyard” (NIMBY) will clearly reflect this. Typically, they are the postponed constructions of Beijing Liulitun waste incineration power plant project in 2006, the site dispute of Guangzhou Panyu waste incineration power plant in 2009 and the Hubei Xiantao waste incineration incident in 2016 [7]. Xu M. and Lin B. designed and carried out a survey on public’s perception towards waste incineration power plants and willingness to pay for the avoidance of the plants in their neighborhoods in Beijing, Shanghai, Guangzhou, and Shenzhen [8]. Linlin, Sun et al. examined the issues of the causes of and strategies of the NIMBY conflict management among the stakeholders. The results of this study show that public participation, EIA (Environmental impact assessment) and the gap between the policy making and the fast city development are the main issues. Tough stability maintenance measures intensified public opposition to the NIMBY facility [9]. Yang et al. Integrated Bayesian network structure discovery and co-word analysis into a qualitative analysis, searched data and key factors from a literature search engine with specific themes were used for structure learning, the result confirmed that the Bayesian network model can help government find the path to transform waste NIMBY crisis, which provided a theoretical basis for understanding the transformation of waste NIMBY crisis in China [10]. Based on this, this paper will further apply relevant theories to answer “why does the waste NIMBY crisis happen?” “How does the waste NIMBY crisis evolve?” Additionally, the scientific validity of the model is verified by a case study.

As a typical social crisis, NIMBY crisis has brought great pressure on social stability. It is necessary to explore the influencing factors of the behavior of NIMBY crisis and understand why the public fight against it [11]. When it comes to this, there is a question, namely, why is the NIMBY crisis becoming more and more intense in the context of China? This question can be understood from two aspects: firstly, why does NIMBY crisis generally from individual protests to group conflicts? Secondly, why is the NIMBY crisis frequently performed in various places of the country? Essentially, this requires the explanation of the causes and evolution mechanism of the NIMBY crisis [12]. The literature review found that the negative externality of the facilities [13,14], political factor [15,16], social trust [17,18], equity factor [19,20] and risk perception [21,22] were important factors for the formation of NIMBY crisis.

NIMBY crisis has the characteristics of high frequency, wide influence and large number of participants. Hence, public protests are increasingly escalating into mass incidents [23]. On the other hand, due to the complexity and random disturbance of the evolution process of NIMBY crisis, the existing research should not be limited to the analysis of events in deterministic environment but explored in a more real uncertain environment [24].

The decision-making model for “prediction–response, ” which is just to take measures by constructing case scenarios based on empirical rules and historical events, cannot meet the demands of real-time decision making in handling emergencies [25]. In recent years, in order to analyze and predict the evolution path and future development trend of emergencies, scholars at home and abroad have proposed corresponding research schemes and scenario deduction models for different types of emergencies. Sheng designed a method for extracting key elements of emergency scenario network. The method took emergency sources, emergency evolution and emergency response as the main body of scenario network, and based on this, the paper make further efforts to selected four types of key elements of scenario network [26].

From the perspective of critical crisis incidents and crisis evolution chain, evolution characteristics and risk control of explosive crisis in coastal nuclear power plant have been analyzed based on the disaster evolution network analysis theory. The results show that the nuclear leakage is a critical crisis in coastal nuclear explosion disaster system [27]. Environmental pollution events are researched to explore the causes of environmental pollution incidents and solve the problem derived from environmental pollution events. The environmental pollution incidents and relevant conflicts of interest are studied by using evolutionary game theory. And when there exists information interaction among groups and public opinion guidance from supervision department, the evolution characteristics of environmental pollution even are displayed [28]. Liu built up a signal game model considering the fixed signal costs and proportional signal costs respectively, which provides a coordinated mechanism of social conflicts to avoid making situation worse [29]. Wang et al. studied the strategy selection process of two types of social groups, i.e., the social powerful group and the social vulnerable group, based on evolution game theory, the paper establishes the evolutionary game model of unexpected incidents involving mass participation under the scenario with or without the higher levels of government’s punishment mechanism, and analyzed the behavior strategy stability of the two heterogeneous groups [30]. From the perspective of scenario evolution, Huang et al. designed the system structure of event scenario evolution as a hierarchical network structure of hazard factors, key hazard-affected bodies, and derivative events [31].

In summary, scholars at home and abroad have applied different analysis methods to study the influencing factors of waste NIMBY crisis, and the evolution trend of emergencies were revealed. However, there is still a lack of a systematic theoretical framework for the scenario evolution of waste NIMBY crisis. In addition, scenario deduction was a dynamic process, and Bayesian network was not enough to reflect its state transition process over time. Based on this, the rest of the paper was organized as follows. Section 2 described the relevant methods and models used in this paper, as well as the process of data collection. Section 3 introduced the logic thinking of the research, firstly, factors of waste NIMBY crisis scenario evolution were revealed. Next, scenario evolution law of waste NIMBY crisis was discovered. Finally, scenario evolution based on Dynamic Bayesian Network was constructed. Section 4 took the waste NIMBY crisis in Xiantao, Hubei Province as the case study, and the dynamic scenario evolution network of it was visually displayed by Netica (Norsys Software Corp., Canada). The discussion and conclusions were detailed displayed in Section 5.

## 2. Methods and Data

### 2.1. Grounded Theory

In this study, we will employ a qualitative research methodology using Grounded Theory (GT) method. GT is an interpretive enquiry method that can be used in research that aims to build a theory through the collection and analysis of empirical data. GT has been argued as a unique research method where theories are generated inductively which are ‘grounded’ in data and not derived deductively from the existing theory [32]. It should also be noted that in GT data gathering and analyses phases are done concurrently and systematically using constant comparison method. The expected outcome of GT study is an emerging theory, which is derived from data, not from inferences of existing theories. Furthermore, the uniqueness of GT method is that in order to generate a theory, a flexible and creative research process is highly needed [33], whereby revision processes are done simultaneously and also guided by writing of memo during the analysis. The analysis of grounded theory is mainly carried out through three types of coding, namely, open coding, axial coding and selective coding [34].

There are three main reasons why this paper chooses GT to analyze the influencing factors of waste NIMBY crisis:

- GT emphasizes the construction of theory, which is helpful to provide a comprehensive interpretation for a certain phenomenon, consequently, it is suitable for analyzing the influencing factors of waste NIMBY crisis;
- Among the existing research results on waste NIMBY crisis, there are more qualitative studies, which provide abundant theoretical basis for coding analysis;
- GT can comprehensively analyze the literature from different research perspectives, which is conducive to breaking the limitation of single research perspective, so as to comprehensively discover the influencing factors of waste NIMBY crisis.

### 2.2. Dynamic Bayesian Networks

The limitations of using Bayesian Networks (BNs) are that no cycle must be formed in their cause-and-effect paths; since this will lead to issues with feedbacks and will produce incorrect results. A Dynamic Bayesian Networks (DBN) can be substituted since it can model the data time delays and allows the creation of cyclic networks. [35]. In view of the time dependence of scenario evolution in different stages, it is necessary to use DBN on time series to process time series data. DBN is a kind of modeling and reasoning tool for dynamic system developed in recent years. It adds time factor on the basis of static Bayesian network, which makes the process of event reasoning have continuity, consequently, more in line with the objective requirements. The application of DBN model discussed in recent studies are briefly presented below. Khakzad et al. used DBN method to assess the performance of fire protection systems during domino effects [36]. Kammouh et al. created a probabilistic framework in order to evaluate the resilience of engineering systems using conventional and DBN [37]. At a certain moment, the state attribute of current situation is produced by the joint action of the situation state of the previous stage, the external situation of this stage, and emergency management. Because of the Markov characteristics of scenario representation, it is suitable to use DBN to describe the scenario deduction.

Suppose x is the causal set, or parent node set. Set X contains *n* elements, each of which is denoted as x_i_, x_i_∈ X (i = 1, 2, …, *n*); y is the result set in causality or called the set of child nodes. Set Y contains *n* elements, each of which is marked as y_i_, y_i_ ∈ Y (I = 1, 2, …, *n*). The total probability formula is the probability of the result when the cause is known, as shown in Equation (1):

(1) $$p(y)=p(x_{1}y)+p(x_{2}y)+\cdots p(x_{n}y)$$

According to Equation (1), the total probability formula is the calculation result of known causes and causality. Bayesian network is a directed acyclic graph composed of variable nodes and directed edges of connected nodes. It supports incomplete data reasoning and uncertain reasoning and predict the occurrence probability of incidents. As long as the prior probability of the parent node and the conditional probability from the parent node to the child node are given, the posterior probability of the child node can be calculated. Contrary to the total probability formula, Bayesian formula is to determine the cause of a certain result that has already known to occur, the expression is displayed as Equation (2):

(2) $$p(x_{i}|y)=\frac{p(x_{i})p(y|x_{i})}{p(y)}$$

According to the conditional independence assumption of Bayesian network, if the parent node set of a node is given, then, the node is independent of all its non-descendant nodes, therefore, the joint probability can be expressed as Equation (3):

(3) $$p(x_{1},x_{2},\cdots ,x_{n})=\prod_{i=1}^{n}p(x_{i}|x_{1},x_{2},\cdots ,x_{i-1})$$

**Figure 1:**
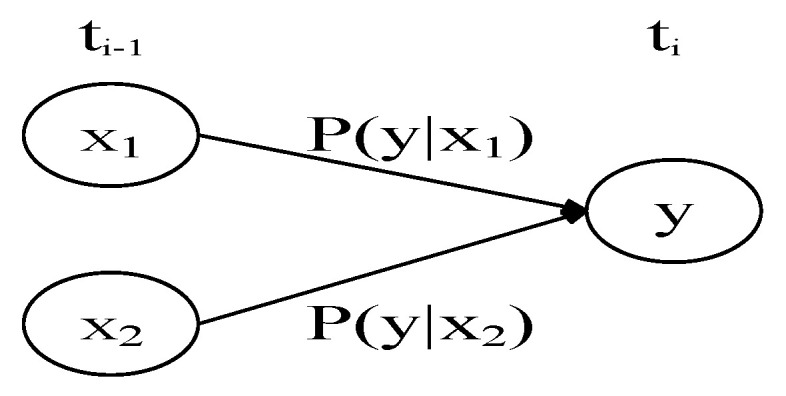
Dynamic Bayesian Network. (Note: X_1_, X_2_ are parent nodes, y is child nodes, and t_i−1_, t_i_. represent different times.).

DBN also conforms to the conditional independence assumption. *x* represents the parent node and y represents the child node (Figure 1).

### 2.3. Data Collection

The incidents of waste NIMBY crisis should be discussed based on the social, economic and political background of each country, so as to improve the theoretical system of localization. Based on this, combined with the purpose of the study, the paper chooses to study domestic waste NIMBY crisis for analysis. In order to ensure the reliability and validity of the study, the paper selects two ways and three successive rounds to obtain case data.

Firstly, determined the typical cases through the web. First of all, with the keywords of “waste NIMBY”, “NIMBY crisis”, “NIMBY risk” and “waste incineration”, the domestic waste NIMBY crisis incidents were widely collected, and case base was established through Baidu, Google, government official websites, government micro-blog and professional websites and forums related to waste NIMBY crisis. Then, the typical case selection were determined by following principles: (a) the selected case had representativeness, that is, the case had great social influence and has been widely concerned by academia, media and society; (b) the types of cases were diversified, and the selected cases were diversified in terms of location and time span; (c) the supporting materials of the cases were comprehensive, including media reports, academic papers, forum posts, blogs, microblogs and publicity Letters, press conferences and other types of materials. Finally, according to the selection principles of typical case, 26 typical cases were selected as the final research samples after repeated study and comparison. The typical cases of waste NIMBY crisis in China were shown in Appendix A.

Secondly, collected and screened typical cases of waste NIMBY crisis based on web data. By using a variety of representative network platforms to conduct comparative analysis, such as Baidu, Google, government official websites, government micro-blog and professional websites and forums related to waste NIMBY crisis. Then, the typical cases database was searched with any combination of words in “NIMBY” and “waste incineration “. The most relevant web pages were selected according to the criteria of accessibility, most relevant content and most sufficient information.

Thirdly, searched and screened the literature of typical cases of waste NIMBY crisis. China National Knowledge Infrastructure (CNKI) is one of the most powerful, up-to-date, comprehensive, and widely used search engines for the analysis of interdisciplinary, peer-reviewed literature in China. Waste NIMBY crisis in China is a specific domestic problem, for which the collected data should reflect local situation, thus as an acknowledged open database, CNKI is suitable to retrieval literature. The logic of literature retrieval was to search keywords of “NIMBY” and any combination of “conflict”, “crisis”, “syndrome” and “incident” respectively, a total of 128 literatures were obtained from CNKI. Then, according to the research content of the literatures, some literatures without case study was deleted, and 97 literatures were preliminarily obtained. Finally, the research results, conclusions or case materials in the literature were carefully read to pay attention to whether there were statements related to waste NIMBY crisis factors. It should be noted that when two studies discussed the same case, it was necessary to judge whether they presented the research results from different aspects. If so, both papers would be selected, otherwise only one of them would be selected. Based on all the above steps, finally, 69 papers were obtained, which met the requirement of at least 10–12 papers for grounded theory analysis.

In brief, the research object of this paper was China’s waste NIMBY crisis incidents in 2006–2019. Among the 26 waste NIMBY crisis cases, 10 cases occurred 10 years ago, and 6 cases occurred five years ago. Due to the timeliness of information and the appointment mechanism of Chinese government officials, it was difficult to directly know formal (legal or governmental) standpoint at that time through interviews and field observation. Consequently, in order to make up for this deficiency, this paper intended to use the grounded theory method based on text analysis to ensure the successful extraction of essential constructs and propositions hidden in the material. On one hand, in the second round of Web data collection process, the authors referred to information from official website, official micro-blog and government report to objectively reflect formal standpoint. On the other hand, in the third round of case screening process, the literatures with in-depth interviews and field observation to obtain first-hand data were reserved and referenced, which contributed to understand the standpoint of the authorities as protagonists.

## 3. Research Design

### 3.1. Factors of Waste NIMBY Crisis Scenario Evolution

**Figure 2:**
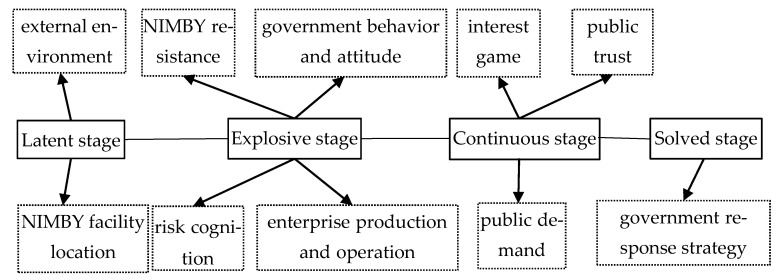
Scenario evolution factors of waste NIMBY (Not-In-My-Back Yard) crisis.

According to the data collection, Nvivo11 software (QSR International, Doncaster Australia) was used to extract, code and classify the data of waste NIMBY crisis. The detailed process was as follows:

- In the open coding stage, all the data information is labeled comprehensively and carefully. Specifically, keywords related to the influence factors of waste NIMBY crisis from the relevant web pages and 69 academic literatures are extracted. In order to improve the consistency of open coding results, two coders conduct precoding analysis under the guidance of a teacher who is familiar with coding analysis. When the consistency of precoding analysis results is good, they can continue to code independently. After detailed open coding analysis of the data, 514 initial concepts are formed after eliminating the repeated, cross and fuzzy sentences, and 43 initial categories are abstracted by inducing the initial concepts. Due to the limitation of space, this paper extracts some results of open coding, an example of this process is shown in Table 1.

- In the axial coding stage, according to the relationship and logical relationship of different initial categories and different levels, the paper classifies and explores them. The core of axial coding is to generalize initial categories with the same connotation with the same abstract concept. For example, public trust in government, experts and environmental assessment can be summarized as public trust. In this way, this paper sums up 10 main categories, including external environment, NIMBY resistance, risk cognition, interest game, public trust, public demand, NIMBY facility location, enterprise production and operation, government response strategy, government behavior and attitude. The result of axial coding is shown in Table 2.

- In the selective coding stage, the purpose was to excavate core category from the main categories and analyze the relationship between categories in the way of story line, so as to establish the substantive theory. The core category identified in this paper is “scenario evolution factors of waste NIMBY crisis”. According to the conclusion of the axial coding, the following diagram (Figure 2), can be constructed by taking the latent stage, explosive stage, continuous stage and solved stage as the “story line”. In the latent stage, external environment and NIMBY facility location were important factors for the occurrence of waste NIMBY crisis. In the explosive stage, risk cognition, NIMBY resistance, government behavior and attitude, enterprise production and operation were key factors to promote scenario evolution of waste NIMBY crisis. In the continuous stage, interest game, public demand and public trust regulate influenced scenario evolution direction of waste NIMBY crisis. In solved stage, government response strategy determined the outcome of scenario evolution of waste NIMBY crisis.

### 3.2. Scenario Evolution Law of Waste NIMBY Crisis

Different from the traditional “typical case”, scenario is not a projection of a specific event, but a collection of numerous similar events and expected risks. Although the number of scenarios is limited, it is widely representative and credible forward-looking. Based on the analysis of scenario evolution factors of waste NIMBY crisis, this paper summarized up three kinds of scenario elements: external situation (E), scenario state (S) and emergency management (M).

**Figure 3:**
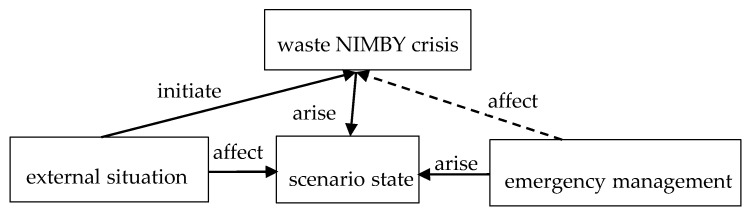
The relationship between the elements of waste NIMBY crisis scenarios.

External situation is the cause of the occurrence of waste NIMBY crisis, and it will continue to promote the development and evolution of waste NIMBY crisis. Scenario state refers to a series of actions taken by the relevant subjects of the waste NIMBY crisis with the accumulation and development of crisis elements, specifically, it is the specific manifestation of MSW NIMBY crisis. Emergency management refers to the actions taken by emergency decision makers in order to control and weaken the loss after the occurrence of waste NIMBY crisis. Integrally, the effectiveness of emergency management affects the evolution direction and intensity of waste NIMBY crisis. This paper portrayed the relationship between external situation, scenario state and emergency management (Figure 3).

It was easy to evolve into a mass emergency if the waste NIMBY crisis was not handled properly. Therefore, the research methods of mass emergency can also be applied to the waste NIMBY crisis. Namely, through mechanism analysis and logical derivation, the scenario evolution law of waste NIMBY crisis can be deduced on the basis of grasping the evolution mechanism of mass emergency.

After the occurrence of the waste NIMBY crisis, it presents a complex network with different evolution paths, and the collected case information is basically unstructured text information, which cannot be directly used for event scenario reasoning. Therefore, in order to facilitate the extraction and decision-making, it is necessary to carry out structural description and processing and transform it into a specific language data structure.

**Figure 4:**
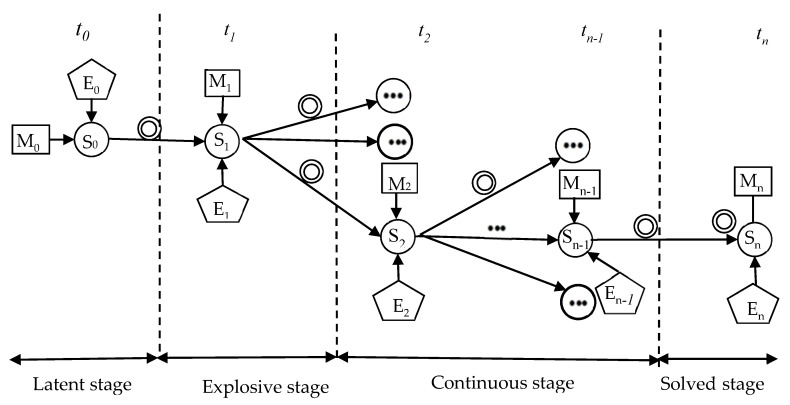
Scenario evolution law of waste NIMBY crisis. (Note: “E” means external situation, “S” means scenario state, “M” means emergency management, “*t*“ represents different times, and “···” means due to the uncertainty of the situation, different new scenarios will appear).

The scenario evolution of waste NIMBY crisis is a dynamic process. Therefore, we should not only clarify the elements of the scenario, but also understand the relationship and interaction between them. Suppose that waste NIMBY crisis experiences *n* scenario transitions from occurrence to disappearance, and the scenario states are recorded as S_0_, S_1_, S_2_, …S_n−1_, S_n_, where S_0_ is the initial scenario state and S_n_ is the disappearing scenario state (Figure 4).

In Figure 4, due to the difference of external situation, scenario state and emergency management, the scenario evolution of waste NIMBY crisis is unpredictable. Therefore, there are many possibilities for the next scenario. Suppose the arrival time t_1_, the situation state is determined by S_0_ and enters to S_1_, under the influence of E_1_ and M_1_, the situation state evolves again, and a new situation state appears; and so on, until the arrival time t_n_, the disappearance scenario S_n_ appears, and the scenario evolution of the waste NIMBY crisis ends.

### 3.3. Scenario Evolution Based on Dynamic Bayesian Network

Firstly, to determine the key node variables of the network. According to the analysis of typical cases, the key element nodes are counted, and combined with expert scoring, the type and value range of node variables are determined.

Secondly, to determine the relationship between network nodes. According to the action principle between node variables and attributes of adjacent nodes, the dependency relationship between nodes is determined. In other words, the directed edge is used to represent the relationship between node variables, and then a complete directed acyclic graph is established according to the development process of incidents scenarios.

Thirdly, to calculate probability of network nodes probability. In order to analyze the scenario evolution more scientifically, two probabilities need to be set in advance: one is the prior probability of scenario node variable without parent node, and the other is the conditional probability of scenario node variable with parent node. Whether the setting of these two probabilities is reasonable or not directly determines the accuracy of the final deduction result. In general, the prior probability is obtained from historical experience or historical statistical data, while the conditional probability is given by expert estimation.

## 4. Case analysis

### 4.1. Case Study of Waste Incineration Power Generation in Xiantao, Hubei Province

In this paper, the waste NIMBY crisis in Xiantao, Hubei Province, was selected as the case study, mainly based on the following considerations. Firstly, it was more difficult to restart the waste incineration power generation project since the waste NIMBY crisis occurred, and the reconstruction of the original site of Xiantao waste incineration power generation project has become a successful case to resolve the “NIMBY effect”; Secondly, the case had a wide influence, which meant the media and academic circles paid more attention to it, consequently, abundant information could be obtained through news reports, Internet, academic papers and other channels. The process of Xiantao waste NIMBY crisis was as follows:

- On 23 June 2016, in Xiantao City, Hubei Province, netizens set up a Wechat group of “Xiantao waste incineration project rights protection” to organize other people to resist the construction of the municipal solid waste incineration power generation project under planning in Xiantao City;
- On 24 June 2016, China xiantao.com released the news that the “Xiantao waste incineration power generation project” officially laid the foundation and started construction, which made the public feel more anxious;
- On 25 June 2016, due to the fact that the location of the “waste incineration power generation project” is too close to the residential area, and the local residents worried that the waste incineration plant would cause pollution problems, some people spontaneously demonstrated in the streets to protest the waste incineration power generation project. Local public security personnel went to the demonstration site to disperse the masses. Violent conflicts broke out between the police and the public, and some residents were injured in the process of the conflict;
- On the evening of 25 June 2016, Xiantao municipal Party committee and municipal government held a press conference to explain the safety and necessity of the project and other relevant issues of public concern;
- On the morning of June 26, 2016, Xiantao municipal Party committee and municipal government successively issued two pieces of news about the project’s postponement. However, after the announcement, the public believed that the saying “to be further evaluated” indicated that the government also lacked confidence in the previous assessment, and the public’s distrust attitude became more and more intense;
- At 12:00 on 26 June 2016, Xiantao municipal Party committee and municipal government gave orders to stop the construction of the project. People’s demands have been resolved and the situation has gradually subsided;
- From November 2016 to May 2017, Xiantao municipal Party committee and municipal government successively organized 19 groups of 2100 people to visit Guangdong, Jiangsu, Zhejiang and other places to inspect solid waste treatment environmental protection industrial parks, waste incineration power generation projects and Yingfeng environmental science and technology group. At the same time, publicity and education work of circular economy industrial park was carried out to solve doubts face to face;
- On 3 May 2017, with the support rate of 99%, Xiantao waste incineration power generation project as the “No. 1 project” of the city was restarted at the original site;
- On 15 April 2018, the waste incineration power generation project was put into trial operation, thus, the waste NIMBY crisis in Xiantao came to an end.

### 4.2. Construction of Dynamic Scenario Evolution Network

Through the case analysis of Xiantao waste NIMBY crisis, on the basis of the analysis of scenario elements, appropriate simplification was made. Then, according to the steps of building Dynamic Bayesian Network, scenario state nodes and their corresponding external environment and emergency management nodes were finally determined.

Concretely, there were eight scenario state nodes, and they were divided into four parts according to the stages of waste NIMBY crisis, for example, in latent stage, the behavior of residents set up a Wechat group of “Xiantao waste incineration project rights protection” to organize other people to resist the construction of the municipal solid waste incineration power generation project under planning was marked as S_0_. Correspondingly, there were seven external environment nodes, the external environment influenced the development of scenario state through different forms of expression. Furthermore, there were seven emergency management nodes under the common affection of scenario state and external environment. The node variables of scenario network were shown in Table 3.

The type of external environment belongs to binary sequential variable. Therefore, its value was set to positive (P) or Negative (N). Moreover, the type of scenario state and emergency management belong to Boolean variable, hence their value was set to true (T) or false (F). Specifically, the type and value set of network node variable were shown in Table 4.

**Figure 5:**
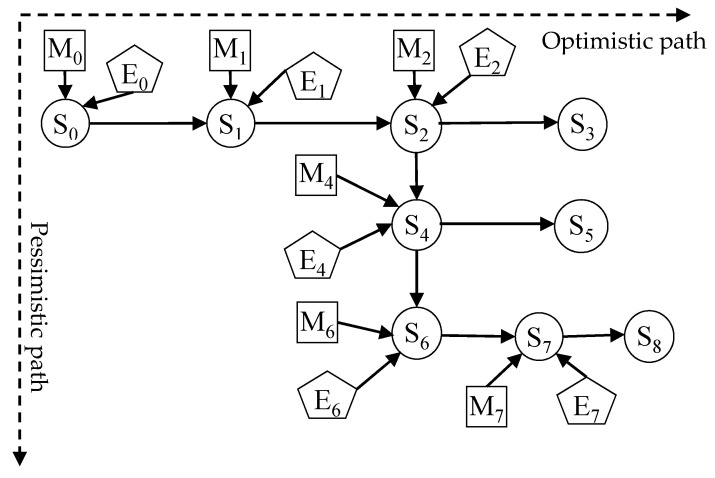
Xiantao waste NIMBY crisis scenario evolution path. (Note: “E” means external situation, “S” means scenario state, and “M” means emergency management.).

After the node variables of scenario network are determined, the relationship between them is expressed by the directed edge. The determination of node relationship is a qualitative process, which is based on the actual case and combined with the evolution law of similar incidents. By analyzing the causal relationship of scenario elements in each stage in the Table 3, Xiantao waste NIMBY crisis scenario evolution path was established (Figure 5). Which contributed to quickly count the scenario nodes that failed to meet the expectation direction on the evolutionary path, found out the path with poor effect of emergency measures, and timely adjusted the emergency measures, so as to make the evolution path of scenario evolve as optimistically as possible.

### 4.3. Scenario Probability Analysis and Calculation

As there are many factors involved in the waste NIMBY crisis, and the evolution is complex, few historical experience data can be obtained for reference, so the expert evaluation and scoring method is mainly used. For accurate scoring results, the detailed scoring rules of network nodes are shown in Table 5.

According to the development and evolution of crisis in different stages, the conditional probability of each node variable was determined based on historical data experience and expert knowledge. The conditional probability of S_0_ node variables was shown in Table 6. Calculation of probability of all nodes were shown in the Appendix B.

**Figure 6:**
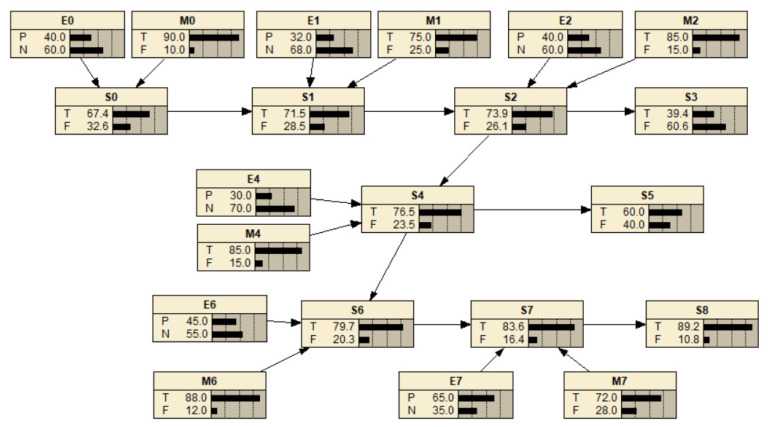
Dynamic scenario evolution network of Xiantao waste NIMBY crisis. (Note: “E” means external situation, “S” means scenario state, “M” means emergency management, “T”means True, “F” means Fales, “P” means “Positive”, and “N“ means “Negative.”).

By analogy, the probability of all scenario nodes in scenario network nodes could be calculated. Ultimately, Figure 6 showed dynamic scenario evolution network of Xiantao waste NIMBY crisis by using the computing software Netica (Figure 6).

## 5. Discussion

As scenario evolution of waste NIMBY crisis was a complex and dynamic process, emergency decision-makers should make emergency decisions and take measures according to the timely situation state, so as to change the current situation state and enter the next scenario state. Due to the uncertainty and dynamic characteristics of the evolution path of waste NIMBY crisis, it was often difficult to take scientific emergency measures. What’s more, in the actual evolution process, emergency management activities may not be able to control the development of the situation, and there would be two directions of incident development and evolution: expectation and un-expectation. if the scenario evolution path of waste NIMBY crisis was consistent with the expected emergency management objectives, in that way, the emergency management of decision-making action was effective, thus, the harm and development trend of the crisis could be effectively controlled. Otherwise, if the scenario evolution path of waste NIMBY crisis deviated from the expected emergency management objective, it could be estimated that the situation was still very serious and even get worse. In general, waste NIMBY crisis was full of uncertainty, and there were multiple possible evolution paths in each scenario, and in the key scenario of incident scenario deduction, the development direction of waste NIMBY crisis was affected by the external environment and emergency measures, which required decision makers to make every step of emergency decision carefully, so as to make the waste NIMBY crisis develop along the expected scenario evolution path as optimistic as possible.

In Figure 6, there were eight scenario states, in which S_3_, S_5_, S_8_ without parent nodes were crisis disappearance scenarios, and other nodes were scenario state nodes. The horizontal dotted arrow indicates the optimistic evolution path of achieving the emergency management objectives under the joint action of external situation and emergency management, such as S_0_→S_1_→S_2_→S_3_. On the other hand, the longitudinal dotted arrow indicates the pessimistic scenario evolution path that the emergency management objectives were not achieved under the interaction of external situation and emergency management, such as S_2_→S_4_→S_6_.

From the latent stage to the solved stage of the waste NIMBY crisis, the scenario states probability of S_0_, S_1_, S_2_, S_4_, S_6_, S_7_ and S_8_ were 67.4%, 71.5%, 73.9%, 76.5%, 79.7%, 83.6% and 89.2%. What’s more, the probability of crisis scenario disappeared increased from 39.4% to 60.0%. On the whole, the probability of scenario state gradually increased with the gradual deterioration of the crisis evolution, which indicated that under the joint action of external situation and emergency management, the situation status of waste NIMBY crisis was evolving in a positive direction. On the other hand, the fluctuation of scenario probability was relatively obvious, which reflected that the external environment tends to be better and emergency management was effective and timely. All in all, the scenario evolution network of Xiantao waste NIMBY crisis was basically accord with the actual crisis development process, which proved the effectiveness and feasibility of the model.

## 6. Conclusions

Due to the complex and irregular evolution of waste NIMBY crisis, it is difficult to take scientific and effective emergency response measures. Therefore, how to reasonably and effectively predict the evolution path and development trend of the incident according to the current state of the incident, and then take scientific and effective emergency response methods to deal with it is particularly important.

In this paper, the authors analyzed the scenario evolution factors of waste NIMBY crisis by utilizing Grounded Theory, on the basis of which, scenarios evolution law of waste NIMBY crisis was obtained. Then, in order to solve the problem of information uncertainty and incompleteness in the process of analyzing the scenario evolution of waste NIMBY crisis, the Dynamic Bayesian Network model was applied to construct the scenario evolution model of waste NIMBY crisis, correspondingly, the construction method of the model was Elaborated. Finally, the paper took Xiantao waste incineration NIMBY crisis as an example, the dynamic scenario evolution network of it was visually displayed by Netica. The simulation results revealed that the calculation results were in line with the actual situation of the crisis evolution, thus, the scenario evolution model based on Dynamic Bayesian Network could effectively deal with the uncertainty and incomplete information of the waste NIMBY crisis. Generally speaking, this paper has certain reference value for the emergency management research of waste NIMBY crisis.

## Figures and Tables

**Table 1:** Open coding of waste NIMBY crisis (excerpt).

Initial Concepts	Case Excerpt
Planning Failure	Individuals were not willing to pay for the government’s planning mistakes.
Government trust	“You can’t even manage the landfill well. How can you manage the incinerator well?”
Government supervision	It was difficult for the government to guarantee the supervision after the approval and completion of the project.
Economic losses	Fruit planted near waste incineration power plants was difficult to sell.
Mass activities	The public took mass activities such as assembly, procession and demonstration to protect their rights.
Right to know	The public had the right to know about major environmental projects. In many cases, they did not know the information of the government.
Government response	In the process of gradually accumulating suspicion, the local government did not respond formally.
Risk aversion	Most people had inexplicable fear and resistance to waste incineration plant.
Interest conflict	For their own interests, the interest collectives did not give up and led to conflicts.
Information opacity	Xiantao municipal government realized that the project information was not transparent, the communication with the public was not sufficient, and the science popularization was not in place.
Government inaction	The inaction of Xiangtan Municipal government also aggravated the disgust and disgust of the public.
Questionnaire procedure	Panyu landscape Bureau said it would start the questionnaire procedure to collect opinions.

**Table 2:** Ten main categories based on axial coding.

Main Categories	Subcategories
External environment	incineration technology environment, social and economic environment
NIMBY (Not-In-My-Back Yard) resistance	means of resistance, resister characteristics, public opinion discussion, public opinion dissemination
Risk cognition	Environmental risk, health risk, economic risks, perceived risk, interest risk, NIMBY syndrome
Interest game	Ally of interest game, conflict of interest, expert standpoint, media standpoint
Public trust	Trust in government, trust in expert, trust in environmental assessment
Public demand	Public participation, information disclosure, public rights, information communication, interest demand, risk compensation, procedural justice
NIMBY facility location	Rationality of site selection, distance between NIMBY facility and house, negative externality of NIMBY facility, public welfare of NIMBY facility
Enterprise production and operation	Enterprise behavior, enterprise reputation, enterprise qualification, enterprise strategy, project income, waste disposal subsidy
Government response strategy	Decision-making model, government governance, emergency measures
Government behavior and attitude	Regulatory mechanism, urban planning, government concept, government behavior

**Table 3:** Scenario network node variables.

Scenario State (S)	External Environment (E)	Emergency Management (M)
Latent stage, set up Wechat rights protection group (S_0_)	Incineration technology environment (E_0_)	Closed decision, not widely canvassed public opinion (M_0_)
Explosive stage, the public parade and demonstration (S_1_)	New media environment (E_1_)	Stability maintaining pressure, kept order by strong arm (M_1_)
Continuous stage, violent conflict between the police and the public (S_2_)	Incineration technology environment (E_2_)	Official response, answered questions and doubts for the public (M_2_)
Scenario disappeared (S_3_)	/	/
Continuous stage, the public remained skeptical (S_4_)	Socio and economic environment (E_4_)	Conducted depth demonstration, suspended the construction of waste incineration plant (M_4_)
Scenario disappeared (S_5_)	/	/
Continuous stage, government deep into trust crisis (S_6_)	New media environment (E_6_)	Respected public opinion and stopped the construction of incineration plant (M_6_)
Continuous stage, People’s demands have not been met (S_7_)	Socio and economic environment (E_7_)	Democratic consultation, carried out publicity and education activities (M_7_)
Solved stage, Reconstructed at the original site (S_8_)	/	/

**Table 4:** Type and value set of network node variable.

Name of Node Variable	Type of Node Variable	Value Set of Network Node Variable
External Environment (E)	Binary sequential variable	{Positive (P), Negative (N)}
Emergency Management (M)	Boolean variable	{True (T), False (F)}
Scenario State (S)	Boolean variable	{True (T, False (F)}

**Table 5:** Detailed scoring rules of network nodes.

Node Name	State	Scoring Criteria
External Environment (E)	Verry good	0.8–1
	Good	0.6–0.8
	Not verry good	below 0.6
Emergency Management (M)	Effective	0.8–1
	General	0.6–0.8
	Ineffective	below 0.6
Scenario State (S)	Verry good	0.8–1
	Good	0.6–0.8
	Not verry good	below 0.6

**Table 6:** Conditional probability of S_0._

External Environment (E_0_)	Emergency Management (M_0_)	True (T)	False (F)
Positive (P)	True (T)	0.65	0.35
Positive (P)	False (F)	0.70	0.30
Negative (N)	True (T)	0.68	0.32
Negative (N)	False (F)	0.75	0.25

P (S_0_ = T)= P (E_0_ = P)* P (M_0_ = T)* P (S_0_ = T|E_0_ = P, M_0_ = T) + P (E_0_ = P)* P (M_0_ = F)* P (S_0_ = T|E_0_ = P, M_0_ = F) + P (E_0_ = N)*P (M_0_ = T)*P (S_0_ = T|E_0_ = N, M_0_ = T) + P (E_0_ = N)*P (M_0_ = F)*P (S_0_ = T|E_0_ = N, M_0_ = F) = 0.40*0.90*0.65 + 0.40*0.10*0.70 + 0.60*0.90*0.68 + 0.60*0.10*0.75 = 0.674.

## Data Availability

The data presented in this study are available on request from the corresponding author. The data are not publicly available due to privacy.

## Appendix A

**Number**	**Waste NIMBY Crisis Incident**	**Time**	**Number**	**Waste NIMBY Crisis Incident**	**Time**
1	Beijing Liulitun	2006.12	14	Anhui Taihu	2018.05
2	Beijing Asuwei	2009.05	15	Jiangxi Jiujiang	2018.04
3	Tianjin Shuanggang	2009.08	16	Hubei Hankoubei	2009.03
4	Tianjin Jixian	2016.06	17	Hubei Guodingshan	2014.03
5	Shanghai Jiangqiao	2008.11	18	Hubei Xiantao	2016.06
6	Shanghai Songjiang	2012.05	19	Hubei Yangluo	2019.06
7	Hebei Qinhuangdao	2009.04	20	Hunan Xaingtan	2014.01
8	Jiangsu Tianjingwa	2006.01	21	Guangdong Panyu	2009.09
9	Jiangsu Wujiang	2009.01	22	Guangdong Huadu	2009.12
10	Jiangsu Wuxi	2011.04	23	Guangdong Boluo	2014.09
11	Zhejiang Yuhang	2014.05	24	Guangdong Zhaoqing	2016.07
12	Zhejiang Haiyan	2016.04	25	Guangdong Yunan	2019.06
13	Anhui Shucheng	2018.05	26	Hainan Wanning	2016.11

## Appendix B

**Calculation of probability of all nodes.**

**Probability Calculation**	**Prior Probability**	**Conditional Probability**
P (S0)	P (E0 = P) = 0.40, P (E0 = N) = 0.60 P (M0 = T) = 0.90, P (M0 = F) = 0.10	P (S0 = T|E0 = P, M0 = T) = 0.65 P (S0 = T|E0 = P, M0 = F) = 0.70 P (S0 = T|E0 = N, M0 = T) = 0.68 P (S0 = T|E0 = N, M0 = F) = 0.75
P (S1)	P (E1 = P) = 0.32, P (E1 = N) = 0.68 P (M1 = T) = 0.75, P (M1 = F) = 0.25	P (S1 = T| S0 = T, E1 = P, M1 = T) = 0.70 P (S1 = T| S0 = T, E1 = P, M1 = F) = 0.80 P (S1 = T| S0 = T, E1 = N, M1 = T) = 0.80 P (S1 = T| S0 = T, E1 = N, M1 = F) = 0.50 P (S1 = T| S0 = F, E1 = P, M1 = T) = 0.75 P (S1 = T| S0 = F, E1 = P, M1 = F) = 0.60 P (S1 = T| S0 = F, E1 = N, M1 = T) = 0.70 P (S1 = T| S0 = F, E1 = N, M1 = F) = 0.65
P (S2)	P (E2 = P) = 0.40, P (E2 = N) = 0.60 P (M2 = T) = 0.85, P (M2 = F) = 0.15	P (S2 = T| S1 = T, E2 = P, M2 = T) = 0.80 P (S2 = T| S1 = T, E2 = P, M2 = F) = 0.85 P (S2 = T| S1 = T, E2 = N, M2 = T) = 0.76 P (S2 = T| S1 = T, E2 = N, M2 = F) = 0.73 P (S2 = T| S1 = F, E2 = P, M2 = T) = 0.54 P (S2 = T| S1 = F, E2 = P, M2 = F) = 0.64 P (S2 = T| S1 = F, E2 = N, M2 = T) = 0.70 P (S2 = T| S1 = F, E2 = N, M2 = F) = 0.72
P (S3)	/	P (S3 = T|S2 = T) = 0.25 P (S3 = T|S2 = F) = 0.80
P (S4)	P (E4 = P) = 0.30, P (E4 = N) = 0.70 P (M4 = T) = 0.85, P (M4 = F) = 0.15	P (S4 = T| S2 = T, E4 = P, M4 = T) = 0.78 P (S4 = T| S2 = T, E4 = P, M4 = F) = 0.72 P (S4 = T| S2 = T, E4 = N, M4 = T) = 0.80 P (S4 = T| S2 = T, E4 = N, M4 = F) = 0.65 P (S4 = T| S2 = F, E4 = P, M4 = T) = 0.70 P (S4 = T| S2 = F, E4 = P, M4 = F) = 0.60 P (S4 = T| S2 = F, E4 = N, M4 = T) = 0.75 P (S4 = T| S2 = F, E4 = N, M4 = F) = 0.80
P (S5)	/	P (S5 = T|S4 = T) = 0.60 P (S5 = T|S4 = F) = 0.60
P (S6)	P (E6 = P) = 0.45, P (E6 = N) = 0.55 P (M6 = T) = 0.88, P (M6 = F) = 0.12	P (S6 = T| S4 = T, E6 = P, M6 = T) = 0.79 P (S6 = T| S4 = T, E6 = P, M6 = F) = 0.75 P (S6 = T| S4 = T, E6 = N, M6 = T) = 0.80 P (S6 = T| S4 = T, E6 = N, M6 = F) = 0.85 P (S6 = F| S4 = F, E6 = P, M6 = T) = 0.78 P (S6 = T| S4 = F, E6 = P, M6 = F) = 0.90 P (S6 = T| S4 = F, E6 = N, M6 = T) = 0.80 P (S6 = T| S4 = F, E6 = N, M6 = F) = 0.82
P (S7)	P (E7 = P) = 0.65, P (E7 = N) = 0.35 P (M7 = T) = 0.72, P (M7 = F) = 0.28	P (S7 = T| S6 = T, E7 = P, M7 = T) = 0.78 P (S7 = T| S6 = T, E7 = P, M7 = F) = 0.85 P (S7 = T| S6 = T, E7 = N, M7 = T) = 0.90 P (S7 = T| S6 = T, E7 = N, M7 = F) = 0.93 P (S7 = T| S6 = F, E7 = P, M7 = T) = 0.84 P (S7 = T| S6 = F, E7 = P, M7 = F) = 0.80 P (S7 = T| S6 = F, E7 = N, M7 = T) = 0.81 P (S7 = T| S6 = F, E7 = N, M7 = F) = 0.87
P (S8)	/	P (S5 = T|S4 = T) = 0.90 P (S5 = T|S4 = F) = 0.85

## References

[1] Lu J.W., Zhang S., Hai J., Lei M. (2017). Status and perspectives of municipal solid waste incineration in China: A comparison with developed regions. Waste Manag..

[2] Lu J.W., Xie Y., Xu B., Huang Y., Hai J., Zhang J. (2019). From NIMBY to BIMBY: An evaluation of aesthetic appearance and social sustainability of MSW incineration plants in China. Waste Manag..

[3] Zhang X., Xu J.G., Ju Y. (2018). Public participation in NIMBY risk mitigation: A discourse zoning approach in the Chinese context. Land Use Policy.

[4] Hanna P., Vanclay F., Langdon E.J. (2016). Conceptualizing social protest and the significance of protest actions to large projects. Ext. Ind. Soc..

[5] Ru X. (2020). Cognitive bias of risk communication in NIMBY conflicts and its governance. J. Manag..

[6] Wu Y., Zhai G., Li S., Ren C., Tsuchida S. (2014). Comparative research on NIMBY risk acceptability between Chinese and Japanese college students. Environ. Monit. Assess..

[7] Yang Q., He L., Liu X., Cheng M. (2018). Bayesian-based conflict conversion path discovery for waste management policy implementation in China. Int. J. Conf. Manag..

[8] Xu M., Lin B. (2020). Exploring the “not in my backyard” effect in the construction of waste incineration power plants—Based on a survey in metropolises of China. Environ. Impact Assess. Rev..

[9] Sun L., Yung E.H., Chan E.H., Zhu D. (2016). Issues of NIMBY conflict management from the perspective of stakeholders: A case study in Shanghai. Habitat Int..

[10] Yang Q., Zhu Y., Liu X. (2019). Bayesian-based NIMBY crisis transformation path discovery for municipal solid waste incineration in China. Sustainability.

[11] Gong Z., Peng X., Wang H., Tang Z. (2018). The influencing factors of NIMBY behavior: A qualitative meta-analysis. J. Intel..

[12] Hou G., Wang Y. (2014). Why NIMBY crisis evolutes—An integrated attribution model. J. Pub. Manag..

[13] Pelekasi T., Menegaki M., Damigos D. (2012). Externalities, NIMBY syndrome and marble quarrying activity. J. Environ. Plan. Manag..

[14] Yang X. (2020). Cross-domain environmental NIMBY risk: Scale politics and multilevel governance. Probe.

[15] Nakazawa T. (2016). Politics of distributive justice in the siting of waste disposal facilities: The case of Tokyo. Environ. Politics.

[16] Zhao X. (2014). Government decision factors in NIMBY conflict and the countermeasures. J. Wuhan Univ. Technol..

[17] Chung J.B., Kim H.K. (2009). Competition, economic benefits, trust, and risk perception in siting a potentially hazardous facility. Landsc. Urban Plan..

[18] Xin F. (2018). Government trust in the social amplification of NIMBY risk: From loss to reconstitution. Chin. Pub. Admin..

[19] Xin F., Sun R. (2016). Public participation in environmental governance: A case of partnership and authorized in Jiaxing city. J. Shang Admin. Inst..

[20] Wu Z. (2020). Thinking on the how to face NIMBY conflict in the vision of environmental justice. Dyn. Soc. Sci..

[21] Lv S., Wang Q. (2017). How to promote NIMBY project in dilemma: An analysis based on “Benefit-risk” perception theory. Chin. Pub. Admin..

[22] Chen H., Xing P. (2019). Comparison of Chinese and foreign studies on public participation in NIMBY conflicts: Evolution process, analysis logic and solution strategy. Chin. Pub. Admin..

[23] Heng X., Chen X. (2020). Research on the simulation of the evolution mechanism of NIMBY risk. J. Shanghai Admin. Inst..

[24] Chen H., Lu W., Du L. (2020). Stochastic evolutionary scenario analysis of risk set clustering NIMBY conflicts events. Chin. J. Manag. Sci..

[25] Luo A.C., Chen S.W., Fang C.Y. (2015). Gaussian successive fuzzy integral for sequential multi-decision making. Int. J. Fuzzy Syst..

[26] Sheng Y., Sun Q., Wang Y. (2015). Emergency scenario evolution and extraction method of key elements. J. Safe Sci. Technol..

[27] Chen L., Chen C., Zhao D. (2017). Risk analysis on coastal nuclear power plant based on disaster evolution network. J. Catas..

[28] Zheng J.J., Yan L., Zhang H.Y., He H. (2015). Disposal mechanism of environmental pollution mass incidents based on evolutionary game and optimization theory. Chin. J. Manag..

[29] Liu D., Wang W. (2011). Evolutionary scenario analysis of action priorities for mass emergency in the latent period based on signaling game. J. Pub. Manag..

[30] Wang X., Li Y., Sun H. (2015). Evolutionary game analysis of unexpected incidents involving mass participation based scenario inference. J. Manag. Sci..

[31] Huang W., Wang Q., Ding B., Cao J. (2019). A model based on a fuzzy petri net for scenario evolution of unconventional emergencies. Hum. Cent. Comput..

[32] Hamid W.H.W., Saman M.Z.M., Saud M.S. (2012). Exploring factors influencing the transfer of training using a grounded theory study: Issues and research agenda. Procedia Soc. Behav. Sci..

[33] Tan J. (2010). Grounded theory in practice: Issues and discussion for new qualitative researchers. J. Doc..

[34] Tian Q., Zhang S., Yu H. (2019). Exploring the factors influencing business model innovation using grounded theory: The case of a Chinese high-end equipment manufacturer. Sustainability.

[35] Jafari M.J., Pouyakian M., Khanteymoori A. (2020). Reliability evaluation of fire alarm systems using dynamic bayesian networks and fuzzy fault tree analysis. J. Loss. Prev. Proc. Ind..

[36] Khakzad N., Landucci G., Reniers G. (2017). Application of dynamic Bayesian network to performance assessment of fire protection systems during domino effects. Reliab. Eng. Syst. Saf..

[37] Kammouh O., Gardoni P., Cimellaro G.P. (2020). Probabilistic framework to evaluate the resilience of engineering systems using Bayesian and dynamic Bayesian networks. Reliab. Eng. Sys. Saf..

